# Cytopenia first – hepatitis second: an unusual sequence in aplastic anemia

**DOI:** 10.1002/ccr3.917

**Published:** 2017-04-11

**Authors:** Ferras Alashkar, Daniel Föhring, Ulrich Dührsen, Hideo Andreas Baba, Alexander Röth

**Affiliations:** ^1^ Department of HematologyWest German Cancer CentreUniversity Hospital EssenUniversity of Duisburg‐EssenEssenGermany; ^2^ Institute of PathologyUniversity Hospital EssenUniversity of Duisburg‐EssenEssenGermany

**Keywords:** Aplastic anemia, autoimmune hepatitis, horse antithymocyte globulin, immunosuppressive therapy

## Abstract

To the best of our knowledge, this is the first report of aplastic anemia (AA) preceding autoantibody‐negative autoimmune hepatitis (AIH) with successful treatment of both conditions with the same immunosuppressive regimen, resulting in hematopoietic reconstitution and remission of AIH.

## Introduction

Aplastic anemia (AA) is a rare disorder arising from hematopoietic failure. It is characterized by bi‐ or tricytopenia and a hypocellular bone marrow in the absence of an abnormal infiltrate and no increase in reticulin. In most of the cases, the primary etiology remains unknown, while in <5% AA can be observed following an infection, in particular after hepatitis due to an unidentified pathogen. Patients with AA present with symptoms of anemia, neutropenia, and bleeding complications 1. Depending on disease severity, patients' age, and the availability of a potential HLA‐identical donor, different therapeutic strategies are favored. Immunosuppressive therapy (IST) with antithymocyte globulin (horse ATG) and cyclosporin (CSA) results in a hematologic recovery in 60–70% of patients and is considered the initial standard treatment besides bone marrow transplantation 1.

## Case History

In a 21‐year‐old male, the diagnosis of an acquired, idiopathic nonsevere aplastic anemia (nSAA) was confirmed in October 2012 (ANC [absolute neutrophil count] 1.3/nL, platelets (PLT) 28/nL, reticulocytes (ARC [absolute reticulocyte count] 79.2/nL)), despite stable other hematologic indices: Hb (hemoglobin) 11.8 g/dL, LDH (lactate dehydrogenase) 187 U/L, total serum bilirubin 0.6 mg/dL (10.2 *μ*mol/L), AST (aspartate aminotransferase) 29 U/L, ALT (alanine aminotransferase) 68 U/L, ALP (alkaline phosphatase) 50 U/L. Cytogenetic analysis was unremarkable, revealing a normal karyotype with no evidence of chromosomal abnormalities (46, XY). By that time, a small PNH clone (PNH granulocyte clone size 0.4%) was confirmed by high sensitivity flow cytometry. As the patient was asymptomatic, no specific therapy was indicated, allowing an observational approach to be the treatment of choice.

**Figure 1:**
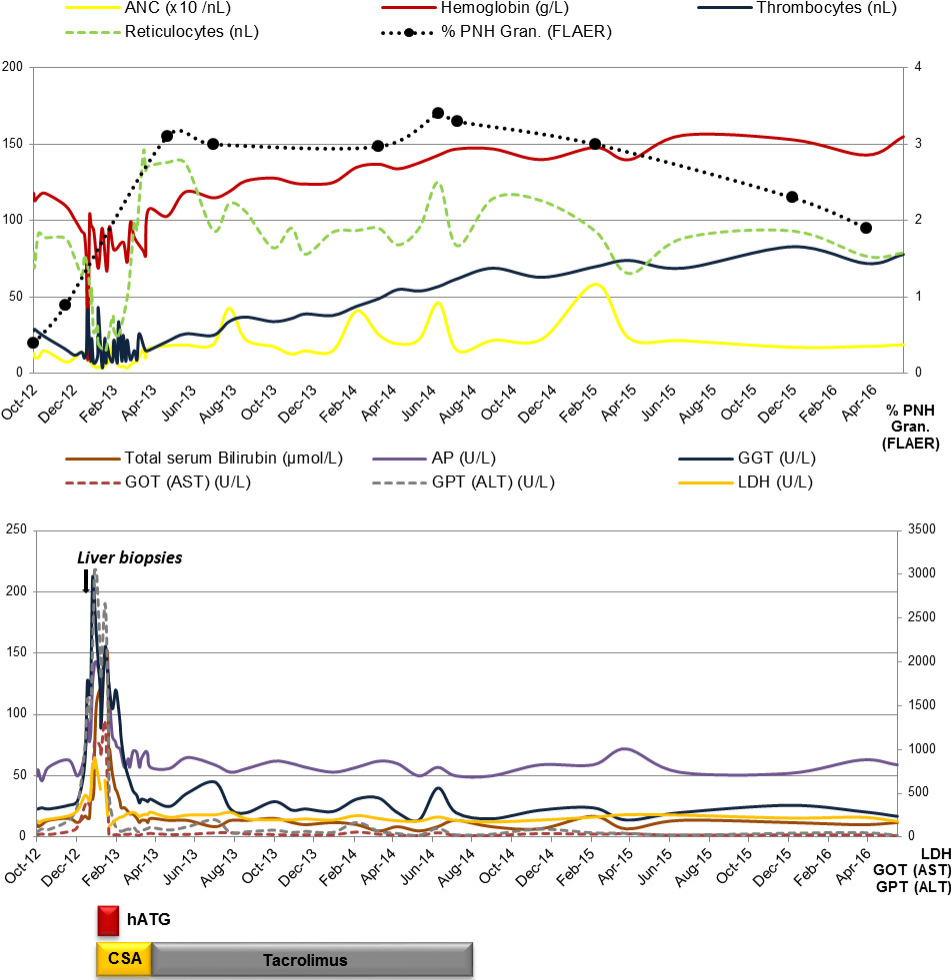
Clinical and laboratory course of a male patient with nonsevere aplastic anemia (nSAA) preceding autoantibody‐negative autoimmune hepatitis (AIH).

**Figure 2:**
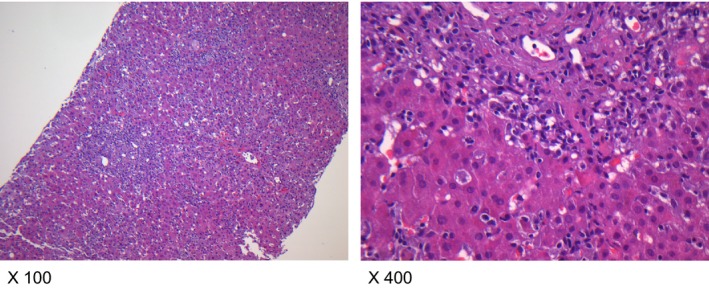
Mild portal fibrosis with prominent portal/periportal and lobular inflammation as well as notable apoptotic hepatocytes in liver biopsy (Magnification: x 100 and x 400).

In December 2012, the patient presented with rapidly progressive elevation of liver enzymes until January 2013 (AST 1324 U/L, ALT 3054 U/L, AST/ALT ratio 0.43, ALP 322 U/L, GGT (gamma‐glutamyl transferase) 212 U/L, hyperbilirubinemia (total serum bilirubin 9 mg/dL (153 *μ*mol/L)), direct bilirubin 0.4 mg/dL), indicating acute hepatic failure (Fig. 1). Abdominal duplex ultrasonography and MRI (magnetic resonance imaging) excluded posthepatic failure, demonstrating mild hepatic steatosis. In addition, transient elastography was unremarkable (5.8 kPA). PCR was negative for hepatotropic viruses, including HSV, VZV, CMV, EBV, and parvovirus B19, excluding a chronic (active) or recently acquired viral or spirochetal hepatitis (L. interrogans antibody titer <1:80). A drug‐induced hepatitis could further be excluded. The subsequent liver biopsies revealed mild fibrosis in combination with periportal and lobular hepatitis and canalicular cholestasis (Fig. 2). Specific autoantibodies for autoimmune hepatitis (AIH), including antinuclear antibodies (ANA: <1:80) and antisoluble liver antigen SLA (SLA: <2RE/mL), were negative. As a consequence of these findings, evidence of AIH monotherapy with prednisone (250 mg for 3 days) was initiated, resulting in no therapeutic benefit. Remarkable was the fact that a progression in PNH clone size up to 3.4% (June 14) was noticed in the presence of AIH. Due to progressive bone marrow failure marked by an increased transfusion requirement for thrombocytes and packed red blood cells (pRBCs) (ANC 0.3/nL, Hb 9.7 g/dL (transfused), PLT 8/nL, ARC45.9/nL, LDH 789 U/L), a bone marrow diagnostic was performed in January 2013, demonstrating a hypocellular bone marrow with no cytogenetic abnormalities confirming severe aplastic anemia (SAA).

Conveying the impression of progressive bone marrow failure, IST with hATG (40 mg/kg BW (body weight) d1‐4) and CSA (5 mg/kg BW) was initiated, resulting in a rapid decline in liver enzymes up to normal values within 5 weeks, hematopoietic reconstitution, and transfusion independence, achieving partial remission (PR) in August 2013 (ANC 4.3/nL, Hb 11.9 g/dL, PLT 34/nL, ARC 111.2/nL, LDH 284 U/L).

A switch in the IST to tacrolimus was indicated due to CSA‐induced hypertensive emergency in March 2013. As hematologic indices were stable for more than 12 months following IST, tacrolimus was tapered down and discontinued by August 2014. On the last day of observation in May 2016, hematologic indices remained stable (ANC 1.8/nL, Hb 15.5 g/dL, PLT 78/nL, ARC 79/nL, LDH 181 U/L, PNH clone size of 1.9%).

## Discussion

Aplastic anemia (AA) is a rare complication of hepatitis (<5%), affecting most often adolescent boys or young men 1. The agent(s) responsible for hepatitis in these cases are not well defined. As signs and symptoms are rather unspecific for hepatitis, patients present with prodromal symptoms including fatigue, myalgia, arthralgia, recurrent episodes of fever, and jaundice 2–3 months prior to the onset of bone marrow failure.

In none of the reported cases of hepatitis‐associated aplastic anemia (HAAA), AA was described to precede autoantibody‐negative AIH. Therefore, we are able to demonstrate an unusual sequence, as AA being the primary immunological event, rather than the consequence of AIH.

Taking into account that HAAA remains uncommon, the majority of cases have been described as fulminate, with mortality rates up to 85%, making HAAA a challenging treatment decision 2. In ≤7% of the patients with acute presentation, autoantibodies cannot be detected 3. At initial presentation, a hypergammaglobulinemia might be absent, as seen in this patient. In general, patients with AIH respond well to corticosteroids (±azathioprine) resulting in a significant improvement in the majority of patients 4. In cases of treatment failure or drug intolerance, calcineurin inhibitors (CSA/tacrolimus) can be considered as salvage therapies 5, 6. The immunopathogenesis of HAAA being summarized by Rauff et al. concerning lymphocyte variations during this syndrome with the accumulation of activated cytotoxic T cells in the liver, defective monocyte to macrophage differentiation, and decreased circulating levels of interleukin‐1, in addition to response to IST, suggests autoimmunity as the key mechanism 7. The importance of activated CD8‐positive lymphocytes and their cytotoxic effect to hematopoietic progenitor cells has been described by Kagan et al. 8. As a possible antigenic overlap between hematopoietic stem cells and the liver compartment can be speculated 9, IST with the same IS strategy suggests a reasonable therapeutic approach for both immune‐mediated AA and AIH.

Interferon‐gamma, a mediator of hematopoietic suppression, being secreted by activated CD8‐positive T cells, might plays an additional role 10.

## Authorship

FA: performed the research, designed the research study, analyzed the data, and wrote the manuscript. DF: performed the research and analyzed the data. UD: analyzed the data and wrote the manuscript. HAB: performed the research, contributed essential reagents or tools, analyzed the data, and wrote the manuscript. AR: performed the research, designed the research study, analyzed the data, and wrote the manuscript.

## Conflict of Interests

The authors have indicated that they have no conflict of interests regarding the content of this article.
